# Adverse childhood experiences and the risk of endometriosis—a nationwide cohort study

**DOI:** 10.1093/humrep/deaf101

**Published:** 2025-06-11

**Authors:** Marika Rostvall, Cecilia Magnusson, Kristina Gemzell-Danielsson, Kyriaki Kosidou, Johanna Sieurin

**Affiliations:** Department of Global Public Health, Karolinska Institutet, Stockholm, Sweden; Karolinska University Hospital, Stockholm, Sweden; Department of Global Public Health, Karolinska Institutet, Stockholm, Sweden; Centre for Epidemiology and Community Medicine, Region Stockholm, Stockholm, Sweden; Karolinska University Hospital, Stockholm, Sweden; Department of Women’s and Children’s Health, Karolinska Institutet, Stockholm, Sweden; Department of Global Public Health, Karolinska Institutet, Stockholm, Sweden; Centre for Epidemiology and Community Medicine, Region Stockholm, Stockholm, Sweden; Department of Global Public Health, Karolinska Institutet, Stockholm, Sweden; Centre for Epidemiology and Community Medicine, Region Stockholm, Stockholm, Sweden

**Keywords:** cohort study, childhood maltreatment, childhood adversity, child abuse, dysmenorrhea, endometriosis, epidemiology

## Abstract

**STUDY QUESTION:**

Is childhood adversity associated with the subsequent diagnosis of endometriosis?

**SUMMARY ANSWER:**

Childhood adversities, including parental substance abuse, parental intellectual disability, parental psychiatric disorder, having a teenage parent, child welfare intervention, parental separation, residential instability, receiving public assistance, exposure to violence, and parental exposure to violence were linked to a higher risk of endometriosis, and the risk increased with increasing number of adversities.

**WHAT IS KNOWN ALREADY:**

The etiology of endometriosis is still not fully explained and there is a need for identification and greater understanding of risk factors. A few studies have previously demonstrated an association between self-reported childhood abuse and an increased risk of endometriosis.

**STUDY DESIGN, SIZE, DURATION:**

This prospective cohort study included all women born in Sweden from 1974 to 2001 using data from a linkage of several national registers. Women who died, emigrated, or received an endometriosis diagnosis before age 15 years and women who were adopted were excluded, leaving 1 316 946 women in the analytical sample. We identified 24 311 women with endometriosis based on ICD-codes registered by a health care professional. Childhood adversities were identified through a variety of different registers. Univariable and multivariable Cox proportional hazards regression models were used to estimate hazard ratios between experiencing adversities and subsequent endometriosis diagnosis.

**MAIN RESULTS AND THE ROLE OF CHANCE:**

All the examined adversities, except familial death, were associated with an increased risk of later endometriosis diagnosis. Among the adversities associated with endometriosis risk, the strongest association observed was for exposure to violence (HR = 2.38, 95% CI 1.89–2.99) and the weakest association observed was for having a teenage parent (HR = 1.20, 95% CI 1.13–1.27). Having experienced any adversity was significantly associated with an increased risk for endometriosis diagnosis (HR = 1.20 95% CI 1.17–1.24) and the risk increased with increasing number of adversities, with an up to 60% increase in risk among those who had five or more adversities (HR = 1.61 1.37–1.88). Adjustment for covariates did not substantially influence the results.

**LIMITATIONS, REASONS FOR CAUTION:**

Since endometriosis is widely underdiagnosed there might be many false negatives in the sample, which may introduce bias if differential.

**WIDER IMPLICATIONS OF THE FINDINGS:**

The results of our study suggest that early life adversity is associated with an increased risk of being diagnosed with endometriosis. This finding could help guide further etiological research. It also strengthens the already large amount of evidence showing that childhood adversity has profound consequences for future health, and that there is a need for effective policies to protect children and support parents. Additionally, clinicians might need to be aware of childhood adversity as a potential risk factor for endometriosis development, and make sure to offer a thorough gynecological evaluation in individuals who have experienced childhood adversities and present with pelvic pain or dysmenorrhea.

**STUDY FUNDING/COMPETING INTEREST(S):**

Karolinska Institutet and Region Stockholm supported this work. The funding sources had no direct involvement. K.G.-D. reports honoraria for ad hoc participation as invited speaker or expert for Organon, Bayer, Gedeon Richter, ObsEva, Addeira, Exelgyn/Nordic, Exeltis, Cirqle, and Natural Cycles. The remaining authors report no conflict of interest.

**TRIAL REGISTRATION NUMBER:**

N/A.

## Introduction

Endometriosis is a benign but potentially debilitating gynecological disorder where endometrial-like tissue implants outside the uterus, most often in the pelvic region. Women with endometriosis have increased risks for dysmenorrhea and infertility as well as chronic pain. Approximately 10% of women of reproductive age are believed to be affected, although underdiagnosis is common and considerably fewer women are diagnosed and treated (Zondervan *et al.*, 2020). The etiology of endometriosis remains poorly understood, but genetic, immunological, hormonal, and environmental factors have been suggested to contribute. Several pathophysiological pathways have been proposed, whereof retrograde menstruation is considered among the more plausible (Sampson, 1927). While 90% of menstruating women may experience retrograde menstruation, only a fraction develop endometriosis. A dysfunctional immune system that allows the endometriosis lesions to implant and develop outside the uterus might be a contributing factor (Symons *et al.*, 2018).

Adverse childhood experiences (ACEs) are potentially traumatic events or experiences that occur in childhood or adolescence. The concept also includes aspects of the child’s environment that can undermine their sense of safety, stability, and bonding. ACEs such as parental separation, residential instability, and experiencing violence are very common (Giano *et al.*, 2020) and have been shown to increase the risk of many negative health outcomes, including ischemic heart disease, cancer, mental disorders, and diabetes (Felitti *et al.*, 1998; Bellis *et al.*, 2015; Juwariah *et al.*, 2022). Recently, ACEs have been proposed as a possible risk factor for endometriosis, though the evidence remains limited and conflicting. A study in mice found that early-life adversity accelerated the development of endometriosis lesions (Long *et al.*, 2020), while a few epidemiological studies have shown an increased incidence of endometriosis in women reporting childhood trauma (Tietjen *et al.*, 2010; Harris *et al.*, 2018; Liebermann *et al.*, 2018). However, other studies found no significant association between ACEs and endometriosis (Schliep *et al.*, 2016; Bourdon *et al.*, 2023), although one did report a link between childhood sexual abuse and pelvic pain (Bourdon *et al.*, 2023).

There are several potential explanations for an association between ACEs and an increased risk for endometriosis. One is immune dysregulation (Symons *et al.*, 2018), a potential consequence of ACEs (Wong *et al.*, 2022). Another possible explanation is increased pain sensitization in individuals exposed to ACEs, which may lead to a higher likelihood of seeking medical attention. The pain mechanisms in endometriosis are complex and not fully understood (Gruber and Mechsner, 2021), and the amount of visible endometriosis lesions correlates little with pain severity (Maddern *et al.*, 2020). ACEs have been linked to an increased risk for chronic pain (Bussieres *et al.*, 2023), potentially through pain sensitization, a process where the central nervous system undergoes changes that make it more sensitive to pain. ACEs have also been linked to an increased risk for psychiatric disorders, common comorbidities in endometriosis (Gao *et al.*, 2020b), which can alter pain perception (Kim *et al.*, 2022).

Still, the epidemiological evidence on the association between ACEs and endometriosis remains limited, as few studies have explored this relationship. Previous studies have in general been small, relied on self-reported ACEs, and no previous studies are conducted on the general population (Tietjen *et al.*, 2010; Schliep *et al.*, 2016; Harris *et al.*, 2018; Liebermann *et al.*, 2018; Bourdon *et al.*, 2023). Thus, the aim of this nationwide prospective register-based study was to examine the potential association between a broad range of register-based ACEs and subsequent risk of endometriosis.

## Materials and methods

### Study design and study population

**Figure 1.**
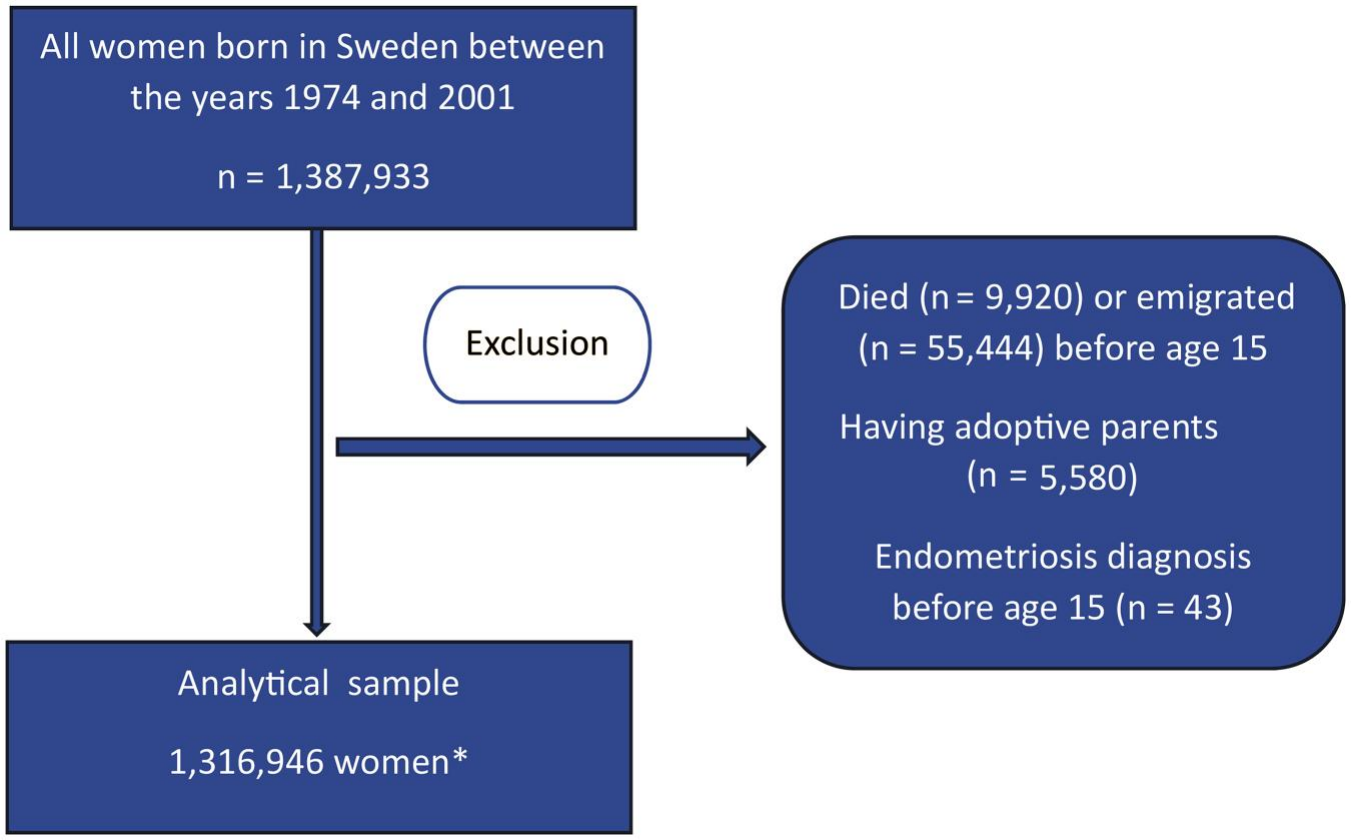
**Flowchart depicting the derivation of the analytical sample.** *The sample for the sensitivity analysis using dysmenorrhea as an outcome only had 1 314 976 participants, due to women who received a dysmenorrhea diagnosis before age 15 years (n = 1977) also being excluded, 7 participants had both diagnoses before age 15 years.

We conducted a register-based nationwide cohort study including all women born in Sweden between the years 1974 and 2001 with two identified biological parents (n = 1 387 933), using data from the Swedish Medical Birth Register (Cnattingius *et al.*, 2023). Study participants were linked to Swedish health and administrative registers using the personal identification number assigned to all Swedish residents upon birth (Ludvigsson *et al.*, 2009). Women who died, emigrated, or received an endometriosis diagnosis before age 15 years and women who were adopted were excluded, leaving 1 316 946 women in the analytical sample, hereafter referred to as the study cohort (Fig. 1). Swedish-born women with at least one adoptive parent were excluded due to the lack of information on adoption dates.

The study participants were followed in the registers from their 15th birthday until date of endometriosis diagnosis, emigration, death, or end of follow-up (31 December 2020) whichever occurred first.

### Data sources

The *Medical Birth Register (MBR)*: Statistics on pregnancies, labor, and newborns. Covers all pregnancies resulting in childbirth in Sweden since 1973.

*The National Patient Register (NPR)*: Data on diagnoses using the Swedish version of the International Classification of Diseases (ICD) from completed inpatient stays since 1964, with complete coverage since 1987. The NPR also includes hospital-based outpatient care since 2001. While coverage for public outpatient care has remained nearly 100%, coverage for private outpatient care has been lower but has gradually increased over time. By 2011, specialist outpatient care coverage reached ∼80% (Ludvigsson *et al.*, 2011).

*The Longitudinal Integration database for Health Insurance and Labour Market Studies (LISA)*: Detailed data on health insurance, parental insurance, and unemployment insurance at the individual level.

*The Swedish National Register of Measures for Children and Young persons (SNRMCY)*: Information on all children who have been placed in out-of-home care by social services.

*The Total Population Register (TPR)*: Data on all Swedish citizens and information on important life and demographic events such as births, deaths, change in civil status, family relationships, housing, migrations within Sweden as well as to and from other countries.

*The Cause of Death Register (CDR)*: Data on all deaths of people registered in Sweden since 1961.

### Assessment of endometriosis

Endometriosis cases were defined by the presence of a main or secondary diagnosis of endometriosis in the NPR using the ICD-codes 617 (ICD-9) and N80 (ICD-10). Endometriosis diagnoses in the NPR have previously been validated and found to have a positive predictive value of 97.8% (Ludvigsson *et al.*, 2011).

### Assessment of dysmenorrhea

To address underdiagnosis and diagnostic delays in endometriosis, we defined an alternative outcome for sensitivity analyses, that encompassed endometriosis, dysmenorrhea, or both. Dysmenorrhea was defined by the presence of a main or secondary diagnosis of dysmenorrhea in the NPR using the ICD-codes 625D (ICD-9) and N94.5 and N94.6 (ICD-10). Previous studies have shown that the prevalence of endometriosis in adolescent girls with dysmenorrhea could be as high as 70% (Janssen *et al.*, 2013).

### Assessment of adverse childhood experiences

We defined ACEs using a range of register-based indicators of traumatic events or prolonged distressing conditions within a child’s family or social environment, associated with long-term adverse social or health outcomes: (Wood *et al.*, 1993; Weitoft *et al.*, 2003; Ekeus *et al.*, 2004; Ringbäck Weitoft *et al.*, 2008; Emerson and Brigham, 2014; Li *et al.*, 2014; Raitasalo and Holmila, 2017; Pierce *et al.*, 2020; McKenna *et al.*, 2021; Rebbe *et al.*, 2021).

- *Parental substance abuse:* Defined as at least one parent being diagnosed with substance abuse (ICD-8: 291, 303–304; ICD-9: 291–292, 303–305; ICD-10: F10–F16, F18–F19) as a main or secondary diagnosis before the index person’s 15th birthday, NPR.
- *Parental intellectual disability*: Defined as at least one parent having a diagnosis of intellectual disability (ICD-8: 310–315; ICD-9: 317–319; ICD-10: F70–79) as a main or secondary diagnosis, NPR.
- *Parental psychiatric disorder:* Defined by at least one parent’s hospital admission with a main diagnosis of a psychiatric disorder (ICD-8: 290, 292–301, 305–315; ICD-9: 290, 293–299, 300–302, 306–315, 317–319; ICD-10: F00–F09, F20–F99) (excluding disorders related to substance abuse) before the index person’s 15th birthday, NPR.
- *Familial death*: Defined as the death of a parent or sibling before the index person’s 15th birthday, CDR.
- *Teenage parent*: Defined as at least one parent being a teenager the year of the child’s birth, TPR.
- *Child welfare intervention:* Defined as at least one placement in out-of-home care before age 15 years, SNRMCY.
- *Parental separation*: Defined as not living with both birth parents at age 16 years, LISA.
- *Residential instability*: Defined as two or more yearly changes in place of residency before age 15 years, TPR.
- *Receiving public assistance*: Defined as the index person’s household having received public social assistance in their 16th year, LISA.
- *Exposure to violence:* Defined as index person being diagnosed with an ICD-code related to interpersonal violence (ICD-8 and 9: 960-969; ICD-10: X85-Y09) before age 15 years, NPR.
- *Parental exposure to violence*: Defined as at least one parent being diagnosed with an ICD-code related to interpersonal violence (ICD-8/9: 960–969; ICD-10: X85-Y09) before the index person’s 15th birthday, NPR.

### Other variables

Data on participants’ county of birth and highest level of education—categorized as primary education or less, secondary education, more than secondary education, or unknown—were obtained from LISA. Data on being born small for gestational age (SGA) were obtained from the MBR.

### Statistical analysis

Participants contributed follow-up time from their 15th birthday, until a registered diagnosis of endometriosis, emigration, death, or until the end of the study in 2020 whichever occurred first. Cox proportional hazard regression models were used to estimate hazard ratios (HRs) and 95% CIs for the associations between ACEs and endometriosis. Age was used as an underlying timescale in all analyses. We calculated descriptive statistics and SDs.

First, each ACE was analyzed separately, using participants unexposed to that specific ACE as the reference group. Second, we analyzed the total ACEs score, incorporating all ACEs significantly associated with an increased endometriosis risk, using those with zero ACEs as the reference group. We performed both univariable and multivariable models, adjusted for birth year, birth county, and SGA in all analyses. Birth year and birth county were included as they may influence both the likelihood of receiving a diagnosis and exposure to or registration of ACEs. SGA has been associated with both an increased risk for endometriosis (Gao *et al.*, 2020a) and maternal low socioeconomic status (Beard *et al.*, 2009). Low childhood socioeconomic status is a major epidemiological risk factor for ACEs (Walsh *et al.*, 2019). Thus, SGA may serve as a proxy for parental factors that could influence the risk of both endometriosis and ACEs.

We performed several sensitivity analyses to assess the robustness of our findings. First, we examined the impact of alternative outcome definitions. As underdiagnosis and diagnostic delays are common in endometriosis (Hudelist *et al.*, 2012) and it is common that women with endometriosis initially receive a dysmenorrhea diagnosis (Janssen *et al.*, 2013) we defined an alternative outcome including a diagnosis of endometriosis, dysmenorrhea, or both. In these analyses, the first date of diagnosis of either dysmenorrhea or endometriosis was used as the end of follow-up for the cases. We also examined if the results differed when only including cases with endometriosis as the main diagnosis at healthcare visits. Next, we included all ACEs in a single model to assess their independent effects. Further, we examined if the association of ACEs defined according to parental characteristics differed depending on the sex of the parent. Given the high median age for endometriosis diagnosis, we repeated the analyses in a subsample of women born 1974–1980 who were older at the end of follow-up and more likely to be diagnosed.

We used SAS 9.4 to perform all analyses.

### Ethics approval

The Swedish Ethical Review Authority (DNR 2020-05514) gave ethical approval for the registry-based analysis and waived the need for informed consent.

## Results

There were 1 316 946 women in the final study sample, aged 32 years on average by the end of the study, who contributed a total of 22 516 511 person-years of follow-up. Overall, 1.9% of the study participants were diagnosed with endometriosis during follow-up (n = 24 311). The mean age at first endometriosis diagnosis was 29 years (SD = 7). More women, 5.3% of the study population, were diagnosed with either endometriosis, dysmenorrhea, or both during follow-up (n = 69 858). Characteristics of the study cohort are shown in Table 1. Parental separation was the most common ACE, experienced by over a third of the study population. The least common ACE was exposure to violence, which affected less than 1% of the study population (Table 1).

**Table 1.** Characteristics of study cohort.

Characteristics (covariates)	Total cohort	Parental substance abuse	Parental intellectual disability	Parental psychiatric disorder	Familial death	Teen parent	Child welfare intervention	Separation	Residential instability	Receiving public assistance	Exposure to violence	Parental exposure to violence
**All**	1 316 946 (100)	54 790 (4)	3221 (0.2)	69 320 (5)	44 366 (3)	39 287 (3)	28 567 (2)	476 869 (36)	43 832 (3)	66 454 (5)	3031 (0.2)	19 422 (2)
**Years of follow up**												
Years	22 516 511	868 830	49 946	1 179 964	800 079	774 960	468 229	7 818 240	759 141	1 251 399	35 954	264 391
**Endometriosis during follow up**												
N	24 311	1171	71	1617	863	1061	675	9493	1025	1737	73	348
**Endometriosis and/or dysmenorrhea**												
N	69 858	3552	217	4601	2522	2648	2106	28 300	2959	4782	246	1272
**Mean age at study exit,**												
Years (SD)	32 (8)	30 (8)	30 (8)	32 (8)	33 (8)	34 (8)	31 (8)	31 (8)	32 (8)	31 (8)	26 (6)	28 (8)
**Birth year**												
1974–1980	319 887 (24)	10 893 (20)	219 (19)	16 974 (24)	12 360 (28)	15 648 (40)	6169 (22)	99 584 (21)	11 155 (25)	21 356 (32)	152 (5)	2572 (13)
1981–1987	314 450 (24)	11 509 (21)	244 (21)	16 472 (24)	11 733 (26)	9177 (23)	6563 (23)	110 397 (23)	10 886 (25)	18 274 (28)	227 (8)	2930 (15)
1988–1994	383 004 (29)	16 585 (30)	373 (32)	19 830 (29)	12 086 (27)	8916 (23)	8511 (30)	15 ,039 (32)	13 496 (31)	16 452 (25)	1253 (41)	5841 (30)
1995–2001	299 605 (23)	15 803 (29)	345 (29)	16 044 (23)	8187 (18)	5546 (14)	7324 (26)	11 ,849 (24)	8295 (19)	10 372 (16)	1399 (46)	8079 (40)
**Attained education**												
≤Primary	119 008 (9)	10 502 (19)	299 (25)	10 437 (15)	5953 (13)	6804 (17)	8542 (30)	62 924 (13)	7774 (18)	14 148 (21)	884 (29)	3920 (20)
Secondary	517 083 (40)	27 210 (50)	550 (47)	30 509 (44)	18 847 (43)	19 039 (49)	13 521 (47)	21 979 (45)	18 140 (41)	31 917 (48)	1522 (50)	10 168 (52)
>Secondary	671 798 (51)	16 339 (30)	243 (21)	27 499 (40)	19 085 (43)	13 074 (33)	5584 (20)	194 652 (41)	17 424 (40)	19 634 (30)	556 (18)	5061 (26)
Unknown	9057 (>1)	739 (1)	89 (8)	875 (1)	481 (1)	370 (1)	920 (3)	4314 (1)	494 (1)	755 (1)	69 (2)	273 (1)
**Born small for gestational age**	38 334 (3)	2555 (5)	160 (5)	2567 (4)	1760 (4)	1826 (5)	1656 (6)	15 895 (3)	1676 (4)	2772 (4)	120 (4)	829 (4)

All numbers are n (%) unless otherwise specified.

Our results indicated that all examined ACEs, except familial death, were associated with an increased risk of endometriosis diagnosis, with HRs ranging from 1.2 (for having a teenage parent) to 2.4 (for exposure to violence). Adjustment for birth year, birth county, and SGA did not substantially influence the results (Table 2).

**Table 2.** Associations between adverse childhood experiences (ACEs) and diagnosed endometriosis.

		Cases	**Crude** ^2^		**Adjusted** ^3^	
ACEs		**n (IR)** ^1^	HR	(95% CI)	HR	(95% CI)
**Parental substance abuse**	*No*	23 140 (1.07)	1	Reference	1	Reference
	*Yes*	1171 (1.35)	1.31	(1.23–1.39)	1.25	(1.18–1.33)
**Parental intellectual disability**	*No*	24 240 (1.08)	1	Reference	1	Reference
	*Yes*	71 (1.42)	1.38	(1.10–1.75)	1.31	(1.03–1.66)
**Parental psychiatric disorder**	*No*	22 694 (1.06)	1	Reference	1	Reference
	*Yes*	1617 (1.37)	1.29	(1.23–1.36)	1.29	(1.23–1.36)
**Familial death**	*No*	23 448 (1.08)	1	Reference	1	Reference
	*Yes*	863 (1.08)	0.98	(0.91–1.04)	0.99	(0.92–1.06)
**Teenage parent**	*No*	23 250 (1.07)	1	Reference	1	Reference
	*Yes*	1061 (1.37)	1.20	(1.12–1.27)	1.30	(1.22–1.38)
**Child welfare intervention**	*No*	23 636 (1.07)	1	Reference	1	Reference
	*Yes*	675 (1.44)	1.37	(1.27–1.48)	1.34	(1.24–1.45)
**Parental separation**	*No*	14 818 (1.01)	1	Reference	1	Reference
	*Yes*	9493 (1.21)	1.24	(1.21–1.28)	1.19	(1.16–1.23)
**Residential instability**	*No*	23 244 (1.07)	1	Reference	1	Reference
	*Yes*	1025 (1.35)	1.25	(1.18–1.34)	1.26	(1.18–1.34)
**Receiving public assistance**	*No*	22 554 (1.06)	1	Reference	1	Reference
	*Yes*	1737 (1.39)	1.25	(1.20–1.32)	1.30	(1.24–1.37)
**Exposure to violence**	*No*	24 238 (1.08)	1	Reference	1	Reference
	*Yes*	73 (2.03)	2.38	(1.89–2.99)	1.93	(1.52–2.43)
**Parental exposure to violence**	*No*	23 963 (1.08)	1	Reference	1	Reference
	*Yes*	348 (1.32)	1.37	(1.24–1.53)	1.22	(1.01–1.36)

1 IR = Incidence rate, cases/10 000 person years.

2 Adjusted for age by design.

3 Adjusted for birth year, birth county, and being born small for gestational age.

HR, hazard ratio.

There was also a trend of increasing endometriosis risk with increased number of ACEs. Individuals who had experienced any single ACE had a 20% increased risk for endometriosis relative to those with no ACEs (HR = 1.20; 95% CI = 1.17–1.24). Those experiencing five or more ACEs had a 60% increased risk (HR = 1.61; 95% CI 1.37–1.88). Again, adjustment for covariates did not substantially alter the results (Table 3).

**Table 3.** Associations between total amount of adverse childhood experiences (ACEs) and diagnosed endometriosis.

Number of ACEs	**Cases n (IR)** ^1^	**Crude** ^2^ **HR (95% CI)**	**Adjusted** ^3^ **HR (95% CI)**
0	13 176 (0.98)	1 (Reference)	1 (Reference)
1	7186 (1.15)	1.20 (1.17–1.24)	1.16 (1.13–1.20)
2	2496 (1.32)	1.35 (1.30–1.41)	1.33 (1.27–1.39)
3	950 (1.46)	1.50 (1.40–1.60)	1.48 (1.39–1.59)
4	342 (1.45)	1.49 (1.34–1.66)	1.45 (1.30–1.62)
5 or more	161 (1.54)	1.61 (1.37–1.88)	1.57 (1.34–1.84)
*P*-value trend		<0.0001	<0.0001

1 IR = Incidence rate, cases/10 000 person years.

2 Adjusted for age by design.

3 Adjusted for birth year, birth county, and being born small for gestational age.

HR, hazard ratio.

Results from sensitivity analyses using different outcome definitions were similar to the main results, both when including dysmenorrhea (Supplementary Tables S1 and S2) and when only including endometriosis as the main diagnosis (Supplementary Tables S3 and S4). Results from analyses adjusting for all ACEs simultaneously showed that the HRs were attenuated but remained significantly increased for all ACEs except for parental intellectual disability and child welfare intervention (Supplementary Table S5). When exposures were analyzed separately by parental sex, the HRs were similar across parental sex, although they appeared stronger for maternal compared to paternal exposure to violence and for paternal compared to maternal intellectual disability (Supplementary Table S6). In the subsample including only the older women associations remained largely significant and the effect sizes were similar (Supplementary Table S7).

## Discussion

This large population-based cohort study examined the association between a broad range of ACEs and the subsequent risk of endometriosis. We found that all examined ACEs, except familial death in childhood, were associated with an increased risk of endometriosis, which remained after adjustment for birth year, birth county, and SGA. The risk increases generally varied between 20% and 35%, while exposure to violence was associated with an over 2-fold increase in risk. Further, we observed a cumulative effect of ACEs on endometriosis risk, with the risk increasing with increasing number of ACEs, reaching a 60% increased risk for those with ≥5 ACEs. Most ACEs remained significantly associated with increased endometriosis risk after adjusting for all other ACEs, indicating independent effects. Only parental intellectual disability and child welfare intervention were no longer statistically significant, possibly due to low statistical power in the fully adjusted model, as being very rare ACEs.

A major strength of our study is the large and general population-based sample, including all Swedish-born women during a 27-year period. Another major strength is the use of prospectively collected register-based data on exposures and outcomes, which eliminates recall bias, a major concern in retrospective studies based on self-reported data.

Our study also has limitations. While the validity of endometriosis diagnoses in the NPR is high (Ludvigsson *et al.*, 2011), sensitivity is likely low due to underdiagnosis (Zondervan *et al.*, 2020), which may introduce bias, most likely toward no association if non-differential. Furthermore, since ACEs are related to a lower socioeconomic status and a lower likelihood of attaining higher education (Houtepen *et al.*, 2020)—potentially influencing health care utilization and the likelihood of receiving an endometriosis diagnosis(Eggert *et al.*, 2008; Lichtenstein *et al.*, 2023; Westwood *et al.*, 2023)—our results may underestimate the true association. Further, our study sample was relatively young at the end of the study and some women may not have received a diagnosis despite symptoms. Moreover, as our study relied on secondary care diagnoses, we likely captured more severe cases of endometriosis, which may limit the generalizability of our findings to primary care settings and milder cases that might not have sought medical attention. However, in Sweden, endometriosis is predominantly diagnosed within secondary care (Socialstyrelsen, 2019), even when women with suspected endometriosis initially present in primary care. To account for diagnostic delays and undiagnosed cases of endometriosis, we performed a sensitivity analysis including women with diagnosed dysmenorrhea, many of whom are likely to represent undiagnosed endometriosis cases (Janssen *et al.*, 2013), which yielded similar results. Similarly, the use of register-based data underreports violence and abuse that do not lead to attention by the health care system or interventions by social services. On the other hand, our study may capture cases among groups that are typically less responsive to surveys. This limits the direct comparability to previous studies using self-reported data. However, our findings align with prior research (Tietjen *et al.*, 2010; Harris *et al.*, 2018; Liebermann *et al.*, 2018) and contribute to strengthening the overall evidence of a link between ACEs and endometriosis. Finally, we could not distinguish between endometriosis and adenomyosis in our data. However, this is unlikely to impact our results, as adenomyosis is rare, affecting <4% of cases classified under ICD-9 code 617 or ICD-10 code N80 (Gao *et al.*, 2020b) and typically occurs in older women (Morassutto *et al.*, 2016).

To our knowledge, this is the first study to use register-based indicators of ACEs to assess endometriosis risk. Nevertheless, our findings align with previous studies reporting an association between self-reported ACEs such as neglect and physical, sexual and emotional abuse, and risk of endometriosis (Tietjen *et al.*, 2010; Harris *et al.*, 2018; Liebermann *et al.*, 2018). By adopting a broad definition of ACEs, our study expands on previous research by demonstrating that, beyond severe traumatic events, circumstances in childhood that may contribute to distress or a disruption of child’s environment or primary relationships are also associated with an increased risk of endometriosis. One previous study also examined other childhood adversities, such as family members’ drug use and incarceration, but observed no associations (Liebermann *et al.*, 2018). However, this study was relatively small and may have lacked power to detect smaller risk differences. Consistent with prior studies indicating a dose–response relationship (Harris *et al.*, 2018), our results suggest that cumulative ACEs increase the endometriosis risk. We also noted that exposure to physical violence, arguably among the most severe ACEs, had the strongest association with endometriosis risk. There are also previous studies reporting no association between sexual or physical abuse and endometriosis risk (Schliep *et al.*, 2016; Bourdon *et al.*, 2023). However, these studies were small and used comparison groups of women with other gynecological conditions previously linked to childhood adversity (D’Aloisio *et al.*, 2010; Jacobs *et al.*, 2015; Fuentes and Christianson, 2018; Tay *et al.*, 2020), which might explain the observed null associations. However, the study by Bourdon *et al.* found an association between sexual abuse during childhood and severe pelvic pain later in life, irrespective of endometriosis diagnosis.

Several biological mechanisms may underlie the association between ACEs and endometriosis. One hypothesis is through alterations in the immune system which is believed to play a key role in endometriosis development and progression (Symons *et al.*, 2018), as childhood maltreatment has been associated with chronic systemic inflammation and elevated inflammatory markers (Coelho *et al.*, 2014). Supporting this, immunological diseases associated with childhood adversity (Gonzalez, 2013) occur more frequently in endometriosis patients (Sinaii *et al.*, 2002). Childhood adversity has also been suggested to induce persistent sensitization of the central stress response systems, including hypothalamic–pituitary–adrenal axis dysregulation (Tarullo and Gunnar, 2006), and women with endometriosis have been shown to exhibit altered salivary cortisol levels (Quinones *et al.*, 2015). Additionally, a study on mice found an increased risk for endometriosis development in mice who had been separated from their mothers in infancy, further supporting a biological link (Long *et al.*, 2020). Another potential mechanism linking ACEs to a higher endometriosis diagnosis rate is increased pain and pain sensitization. ACEs have been associated with a higher risk of chronic pain conditions, including chronic pelvic pain (Krantz *et al.*, 2019), as well as psychiatric disorders (Danielsdottir *et al.*, 2024), common comorbidities in endometriosis (Gao *et al.*, 2020b), that may amplify pain perception (Kim *et al.*, 2022). Thus, ACEs may contribute to increased pain symptom severity, increasing the likelihood of seeking medical care and receiving a diagnosis, rather than increase the risk of developing endometriosis *per se*.

## Conclusion and future perspectives

In this large population- and register-based cohort study, we observed positive associations between several indicators of childhood adversities and later risk of endometriosis. The risk increased with cumulative adversity. Increased knowledge of potential risk factors for endometriosis might aid clinicians in improving diagnostic accuracy and care of endometriosis patients. Future studies should shed light on the mechanisms underlying these associations.

Our results strengthen the already large amount of evidence showing that childhood adversity has profound consequences for future health and that there is a great need for effective policies to protect children and support parents. It is important that these findings are interpreted with care, considering the complexity of individual circumstances and the risk of increasing stigmatization. Although indicators of ACEs used in this study are selected based on known adverse social and health outcomes, it is important to recognize that these measures serve as proxies for underlying circumstances that may contribute to health risks and should not necessarily be seen as inherently harmful.

Our findings are in line with the increased focus on the importance of a multidisciplinary approach to endometriosis treatment, that may need to include psychological interventions (Fang *et al.*, 2024). Additionally, clinicians might need to be aware of childhood adversity as a potential risk factor for endometriosis development, and make sure to offer a thorough gynecological evaluation in individuals who have experienced childhood adversities and present with pelvic pain or dysmenorrhea.

## Supplementary Material

deaf101_Supplementary_Table_S1

deaf101_Supplementary_Table_S2

deaf101_Supplementary_Table_S3

deaf101_Supplementary_Table_S4

deaf101_Supplementary_Table_S5

deaf101_Supplementary_Table_S6

deaf101_Supplementary_Table_S7

## Data Availability

No data are available. Swedish privacy laws prohibit the authors from making registry data publicly available. The data supporting these findings were used under license and ethical approval for this study. These data can be accessed from respective data holders with appropriate ethical approval and adherence to relevant legislation and data protection protocols.
