# Supplementary material for: Adverse childhood experiences and the risk of endometriosis—a nationwide cohort study
Source: Hum Reprod. 2025 Jun 11;40(9):1735–43. doi: 10.1093/humrep/deaf101 (PMC12408909; doi:10.1093/humrep/deaf101)
Supplement: deaf101_Supplementary_Table_S1 [file deaf101_supplementary_table_s1.pdf]

OXFORD
human reproduction

Human Reproduction, 2025, 1–1
https://doi.org/10.1093/humrep/deaf101
Supplementary Data

Supplementary Table S1. Associations between adverse childhood experiences (ACEs) and diagnosed endometriosis and/or dysmenorrhea.

ACEs		Cases N (IR)[1]	Crude[2] HR[4]	(95% CI)	Adjusted[3] HR[4]	(95% CI)
**Parental substance abuse**	*No*	66 306 (3.10)	1	Reference	1	Reference
	Yes	3552 (4.17)	1.37	(1.32–1.41)	1.27	(1.23–1.31)
**Parental intellectual disability**	*No*	69 641 (3.14)	1	Reference	1	Reference
	Yes	217 (4.44)	1.45	(1.27–1.65)	1.31	(1.14–1.50)
**Parental psychiatric disorder**	*No*	65 257 (3.10)	1	Reference	1	Reference
	Yes	4601 (3.97)	1.28	(1.25–1.32)	1.28	(1.25–1.32)
**Familial death**	*No*	67 336 (3.14)	1	Reference	1	Reference
	Yes	2522 (3.20)	1.01	(0.97–1.05)	1.05	(1.01–1.09)
**Teenage parent**	*No*	67 210 (3.13)	1	Reference	1	Reference
	Yes	2648 (3.47)	1.08	(1.04–1.12)	1.28	(1.23–1.34)
**Child welfare intervention**	*No*	67 752 (3.11)	1	Reference	1	Reference
	Yes	2106 (4.60)	1.49	(1.43–1.56)	1.43	(1.37–1.50)
**Parental separation**	*No*	41 558 (2.86)	1	Reference	1	Reference
	Yes	28 300 (3.68)	1.30	(1.28–1.32)	1.21	(1.19–1.23)
**Residential instability**	*No*	66 767 (3.12)	1	Reference	1	Reference
	Yes	2959 (3.97)	1.27	(1.23–1.32)	1.30	(1.26–1.35)
**Receiving public assistance**	*No*	65 030 (3.10)	1	Reference	1	Reference
	Yes	4782 (3.89)	1.24	(1.20–1.27)	1.36	(1.32–1.40)
**Exposure to violence**	*No*	69 612 (3.14)	1	Reference	1	Reference
	Yes	246 (7.10)	2.48	(2.19–2.81)	1.72	(1.51–1.96)
**Parental exposure to violence**	*No*	68 586 (3.12)	1	Reference	1	Reference
	Yes	1272 (4.91)	1.65	(1.57–1.75)	1.31	(1.24–1.39)

[1] IR = Incidence rate, cases/10 000 person years.
[2] Adjusted for age by design.
[3] Adjusted for birth year, birth county, and being born small for gestational age.
[4] Hazard ratio.
