# Supplementary material for: Adverse childhood experiences and the risk of endometriosis—a nationwide cohort study
Source: Hum Reprod. 2025 Jun 11;40(9):1735–43. doi: 10.1093/humrep/deaf101 (PMC12408909; doi:10.1093/humrep/deaf101)
Supplement: deaf101_Supplementary_Table_S2 [file deaf101_supplementary_table_s2.pdf]

OXFORD
human reproduction
Human Reproduction, 2025, 1–1
https://doi.org/10.1093/humrep/deaf101
Supplementary Data

Supplementary Table S2. Associations between total amount of adverse childhood experiences (ACEs) and endometriosis and/or dysmenorrhea.

Number of ACEs	Cases n (IR)[1]	Crude[2] HR[4] (95% CI)	Adjusted[3] HR[4] (95% CI)
0	37 214 (2.81)	1 (Reference)	1 (Reference)
1	21 135 (3.44)	1.24 (1.22–1.26)	1.16 (1.14–1.18)
2	7216 (3.89)	1.39 (1.35–1.42)	1.36 (1.33–1.40)
3	2740 (4.28)	1.53 (1.47–1.59)	1.49 (1.43–1.55)
4	1062 (4.60)	1.65 (1.55–1.76)	1.60 (1.50–1.70)
5 or more	491 (4.82)	1.74 (1.59–1.90)	1.68 (1.53–1.84)
P-value trend		<0.0001	<0.0001

1 IR = Incidence rate, cases/10 000 person years.
2 Adjusted for age by design.
3 Adjusted for birth year, birth county, and being born small for gestational age.
4 Hazard ratio.
