# Supplementary material for: Adverse childhood experiences and the risk of endometriosis—a nationwide cohort study
Source: Hum Reprod. 2025 Jun 11;40(9):1735–43. doi: 10.1093/humrep/deaf101 (PMC12408909; doi:10.1093/humrep/deaf101)
Supplement: deaf101_Supplementary_Table_S3 [file deaf101_supplementary_table_s3.pdf]

OXFORD
human reproduction

Human Reproduction, 2025, 1–1
https://doi.org/10.1093/humrep/deaf101
Supplementary Data

Supplementary Table S3. Associations between adverse childhood experiences (ACEs) and endometriosis, only including main diagnosis of endometriosis.

ACEs		Cases n (IR)[1]	Crude[2] HR[4]	(95% CI)	Adjusted[3] HR[4]	(95% CI)
**Parental substance abuse**	*No*	19 520 (0.90)	1	Reference	1	Reference
	Yes	991 (1.14)	1.31	(1.23–1.39)	1.26	(1.18–1.34)
**Parental intellectual disability**	*No*	20 451 (0.91)	1	Reference	1	Reference
	Yes	60 (1.20)	1.38	(1.07–1.78)	1.29	(0.99–1.68)
**Parental psychiatric disorder**	*No*	19 128 (0.90)	1	Reference	1	Reference
	Yes	991 (1.14)	1.31	(1.24–1.38)	1.31	(1.24–1.39)
**Familial death**	*No*	19 790 (0.91)	1	Reference	1	Reference
	Yes	721 (0.90)	0.97	(0.90–1.04)	0.98	(0.91–1.05)
**Teenage parent**	*No*	19 631 (0.90)	1	Reference	1	Reference
	Yes	880 (1.14)	1.19	(1.11–1.27)	1.28	(1.20–1.37)
**Child welfare intervention**	*No*	19 939 (0.90)	1	Reference	1	Reference
	Yes	572 (1.22)	1.38	(1.27–1.50)	1.35	(1.24–1.47)
**Parental separation**	*No*	12 462 (0.85)	1	Reference	1	Reference
	Yes	8049 (1.03)	1.25	(1.21–1.28)	1.20	(1.17–1.24)
**Residential instability**	*No*	19 602 (0.90)	1	Reference	1	Reference
	Yes	871 (1.15)	1.26	(1.18–1.35)	1.26	(1.18–1.35)
**Receiving public assistance**	*No*	19 040 (0.90)	1	Reference	1	Reference
	Yes	1453 (1.16)	1.25	(1.18–1.32)	1.29	(1.22–1.36)
**Exposure to violence**	*No*	20 454 (0.91)	1	Reference	1	Reference
	Yes	57 (1.59)	2.14	(1.65–2.78)	1.81	(1.40–2.36)
**Parental exposure to violence**	*No*	20 212 (0.91)	1	Reference	1	Reference
	Yes	299 (1.13)	1.39	(1.24–1.55)	1.22	(1.09–1.38)

1 IR = Incidence rate, cases/10 000 person years.
2 Adjusted for age by design.
3 Adjusted for birth year, birth county, and being born small for gestational age.
4 Hazard ratio.
