# Supplementary material for: Adverse childhood experiences and the risk of endometriosis—a nationwide cohort study
Source: Hum Reprod. 2025 Jun 11;40(9):1735–43. doi: 10.1093/humrep/deaf101 (PMC12408909; doi:10.1093/humrep/deaf101)
Supplement: deaf101_Supplementary_Table_S4 [file deaf101_supplementary_table_s4.pdf]

OXFORD

human reproduction

Human Reproduction, 2025, 1–1
https://doi.org/10.1093/humrep/deaf101
Supplementary Data

Supplementary Table S4. Associations between total amount of adverse childhood experiences (ACEs) and endometriosis only including main diagnosis of endometriosis.

Number of ACEs	Cases n (IR)[1]	Crude[2] HR[4] (95% CI)	Adjusted[3] HR[4] (95% CI)
0	11 087 (0.83)	1 (Reference)	1 (Reference)
1	6097 (0.98)	1.21 (1.17–1.25)	1.17 (1.13–1.21)
2	2094 (1.11)	1.35 (1.29–1.41)	1.33 (1.27–1.39)
3	805 (1.23)	1.51 (1.40–1.62)	1.50 (1.39–1.61)
4	288 (1.22)	1.50 (1.33–1.68)	1.44 (1.28–1.63)
5 or more	140 (1.34)	1.66 (1.41–1.97)	1.61 (1.36–1.91)
P-value trend		<0.0001	<0.0001

[1] IR = Incidence rate, cases/10 000 person years.
[2] Adjusted for age by design.
[3] Adjusted for birth year, birth county, and being born small for gestational age.
[4] Hazard ratio.
