# Supplementary material for: Adverse childhood experiences and the risk of endometriosis—a nationwide cohort study
Source: Hum Reprod. 2025 Jun 11;40(9):1735–43. doi: 10.1093/humrep/deaf101 (PMC12408909; doi:10.1093/humrep/deaf101)
Supplement: deaf101_Supplementary_Table_S5 [file deaf101_supplementary_table_s5.pdf]

OXFORD
human reproduction
Human Reproduction, 2025, 1–1
https://doi.org/10.1093/humrep/deaf101
Supplementary Data

Supplementary Table S5. Associations between adverse childhood experiences (ACEs) and endometriosis, adjusted for all the ACEs in the table.

ACEs	HR	(95% CI)
Parental substance abuse	1.08	1.02–1.15
Parental intellectual disability	1.10	0.87–1.40
Parental psychiatric disorder	1.14	1.09–1.21
Teenage parent	1.08	1.02–1.15
Child welfare intervention	1.07	0.99–1.17
Parental separation	1.18	1.15–1.21
Residential instability	1.12	1.05–1.19
Receiving public assistance	1.13	1.07–1.18
Exposure to violence	2.05	1.63–2.59
Parental exposure to violence	1.14	1.02–1.27

HR, hazard ratio.
