# Supplementary material for: Adverse childhood experiences and the risk of endometriosis—a nationwide cohort study
Source: Hum Reprod. 2025 Jun 11;40(9):1735–43. doi: 10.1093/humrep/deaf101 (PMC12408909; doi:10.1093/humrep/deaf101)
Supplement: deaf101_Supplementary_Table_S6 [file deaf101_supplementary_table_s6.pdf]

OXFORD

human reproduction

Human Reproduction, 2025, 1–1

https://doi.org/10.1093/humrep/deaf101

Supplementary Data

Supplementary Table S6. Associations between adverse childhood experiences (ACEs) and endometriosis, separated by parents' sex.

ACEs		Cases n	Crude[1] HR[3] (95% CI)	Adjusted[2] HR[3] (95% CI)
Parental substance abuse	Mother	386	1.38 (1.25–1.52)	1.25 (1.13–1.39)
	Father	858	1.28 (1.19–1.37)	1.24 (1.16–1.33)
Parental intellectual disability	Mother	40	1.22 (0.90–1.67)	1.22 (0.90–1.67)
	Father	32	1.66 (1.17–2.34)	1.46 (1.01–2.11)
Parental psychiatric disorder	Mother	940	1.27 (1.19–1.35)	1.27 (1.19–1.36)
	Father	747	1.31 (1.21–1.40)	1.32 (1.22–1.42)
Familial death	Mother	163	0.89 (0.76–1.04)	0.88 (0.75–1.03)
	Father	444	1.02 (0.93–1.12)	1.04 (0.94–1.14)
	Sibling	270	0.94 (0.84–1.06)	0.97 (0.85–1.09)
Teenage parent	Mother	886	1.17 (1.10–1.25)	1.29 (1.20–1.38)
	Father	303	1.28 (1.15–1.44)	1.33 (1.19–1.50)
Parental exposure to violence	Mother	126	1.58 (1.33–1.88)	1.38 (1.15–1.65)
	Father	233	1.30 (1.14–1.48)	1.16 (1.01–1.32)

1 Adjusted for age by design.
2 Adjusted for birth year, birth county, and being born small for gestational age.
3 Hazard ratio.
