# Supplementary material for: Adverse childhood experiences and the risk of endometriosis—a nationwide cohort study
Source: Hum Reprod. 2025 Jun 11;40(9):1735–43. doi: 10.1093/humrep/deaf101 (PMC12408909; doi:10.1093/humrep/deaf101)
Supplement: deaf101_Supplementary_Table_S7 [file deaf101_supplementary_table_s7.pdf]

OXFORD

human reproduction

Human Reproduction, 2025, 1–1
https://doi.org/10.1093/humrep/deaf101
Supplementary Data

Supplementary Table S7. Associations between total amount of adverse childhood experiences (ACEs) and endometriosis only including women born 1980 or earlier.

Number of ACEs	Crude[2] HR[3] (95% CI)	Adjusted[3] HR[3] (95% CI)
0	1 (Reference)	1 (Reference)
1	1.13 (1.08–1.19)	1.12 (1.07–1.18)
2	1.26 (1.17–1.35)	1.25 (1.16–1.34)
3	1.43 (1.28–1.59)	1.42 (1.27–1.58)
4	1.40 (1.17–1.68)	1.37 (1.14–1.65)
5 or more	1.52 (1.17–1.96)	1.54 (1.19–1.99)
P-value trend	<0.0001	<0.0001

[1] Adjusted for age by design.
[2] Adjusted for birth year, birth county, and being born small for gestational age.
[3] Hazard ratio.

© The Author(s) 2025. Published by Oxford University Press on behalf of European Society of Human Reproduction and Embryology.
This is an Open Access article distributed under the terms of the Creative Commons Attribution License (https://creativecommons.org/licenses/by/4.0/), which permits unrestricted reuse, distribution, and reproduction in any medium, provided the original work is properly cited.
Human Reproduction, 2025, 00, 1–16
https://doi.org/10.1093/humrep/deaf101
Original Article
